# Description of the Lesch‐Nyhan neurobehavioral disorder and its management through participant observation of three young individuals

**DOI:** 10.1002/jmd2.12100

**Published:** 2020-02-18

**Authors:** Anna Bozano, Alessandra Schiaffino, Alessandra Spessa, Francesca Valeriani, Raimondo Mancinelli, Vanna Micheli, Diego Dolcetta

**Affiliations:** ^1^ LND Famiglie Italiane Genoa Italy; ^2^ University of Surrey Guildford UK; ^3^ Università di Siena and “LND Famiglie Italiane” Siena Italy

**Keywords:** Lesch‐Nyhan behavior (LNB), Lesch‐Nyhan disease (LND), LNB management, self‐injurious behavior (SIB)

## Abstract

**Background:**

Lesch‐Nyhan disease (LND; OMIM 300322), caused by virtually absent hypoxanthine‐guanine phosphoribosyltransferase activity, in its classic form is characterised by hyperuricemia, variable cognitive impairment, severe motor disorder and a characteristic behavioural disorder (Lesch‐Nyhan Behavior, LNB), typically described as self‐injurious behavior (SIB) and “self‐mutilation.” This work focuses on the latter aspect with the aim of exploring and broadening it.

**Methods:**

The participant observation method was used to follow three children diagnosed with LND individually, in different contexts of daily life, always with their usual restraints and in the presence of a caregiver.

**Results:**

60 observational sessions, for over 90 total hours, led to the description of 292 LNBs, interfering with different aspects of life. Harmful behaviors could be classified into different categories, based on the life aspect affected and type of harm provoked. Antecedent conditions, consequent reactions, and emotions of the child and different management of the caregiver were recorded for each LNB. We confirmed that patients normally feel pain. Most common emotional reactions are regret and shock. As a consequence of a LNB, increased anxiety was always recorded, never satisfaction. Caregiver strategies most commonly used to stop the LNB and preventing recurrences are reported and discussed.

**Conclusions:**

We are proposing a wider LNB description, beyond the classical Self‐injurious behavior (SIB), stating that it is widespread and pervasive, involving every facet of the patients' life. Caregivers and operators should be aware that they might face different LNBs, and have to recognize them to find the better way to manage patients.

**SYNOPSIS**

The pathognomonic behavior characterizing the Lesch‐Nyhan disease, previously called self‐injurious behavior (SIB), should be intended in a broader way, involving every aspect of the patient's life (“Lesch‐Nyhan Behavior”). To acknowledge such point could help the management of patients and the comprehension of the disease.

## 1 INTRODUCTION

Lesch‐Nyhan disease (LND, OMIM 300322) is a very rare X‐linked disorder due to deficiency of hypoxanthine‐guanine phosphoribosyltransferase (HPRT). Its classic clinical form, with virtually no residual HPRT activity, involves neurological disorders in neuromotor, cognitive and neurobehavioral areas, the latter being the object of the present study.

The neuromotor disorder presents severe motor disability and dystonia with superimposed basic hypotonia, sometimes accompanied by choreoathetosis or spasticity.^1^, ^2^, ^3^

Cognitive impairment is variable,^4^, ^5^, ^6^ but its evaluation in LND patients is interfered by several disturbing motor and behavioural factors.^7^

The behavioural disorder in LND is typically described in literature with the terms self‐injurious behavior (SIB). However, its peculiar and pathognomonic characteristics that differ from SIB associated with other diseases, such as autism and intellectual disability, are currently recognized in terms of severity and age of onset.^8^, ^9^, ^10^

The neurobehavioral disorder (and in particular self‐injury) is the most vexing problem, as agreed by literature, is poorly controlled pharmacologically and causes extreme stress for families and patients.^2^, ^4^, ^11^ There are presently very few studies concerning this aspect of the disease. The purpose of this article is to collect and systematize more information about the various manifestations of LNB, propose a detailed description, and analyze the effectiveness of management methods.

## 2 METHODS

### 2.1 Participants

#### 2.1.1 Individual 1

Five‐years‐old at the beginning of the study, he carries HPRT1 mutation c.485 + 1G > A, with null erythrocyte HPRT activity. He displays a severe classic form, diagnosed 6 months after birth, with self‐injury onset at 2 years. He has biperidene and risperidone therapy. He uses a very strong physical restraint system, dental guard, arm braces. Speech is very limited and communication is mainly nonverbal. He regularly attends kindergarten, swimming pool, and physical therapy treatments.

#### 2.1.2 Individual 2

Five‐years‐old at the beginning of the study, he carries g.IVS6‐1G>C mutation in intron 6 resulting in a splice site loss. No HPRT activity was detectable in erythrocytes. He presents a classic clinical form diagnosed at 6 months of age, with severe dystonia of the limbs; self‐injury onset was at 2 years. Speech is almost absent. He suffers from dysphagia. He has clonazepam and risperidone therapy. He too uses a very strong physical restraint system, dental guard, constraints to arms and legs. He attends kindergarten and a rehabilitation center.

#### 2.1.3 Individual 3

Twelve years and 6 months old at the beginning of the study. Erythrocytes displayed null HPRT and the self‐injury onset was reported at 5 to 6 years, but LND was only diagnosed at the age of 9. He has carbamazepine, trihexyphenidyl, and baclofen therapy.

He uses a wheelchair with anti‐tip bar and wears gloves. He is severely dysarthric, but able to formulate complex sentences. He regularly attends middle school, swimming pool, and physical therapy treatments.

All the three subjects, who never met, live with their families and attend mainstream schools, with a one to one support teacher. The neuropharmacological therapy did not vary during the study.

Every family was fully informed about the study and written consent was signed before starting.

Additional informed consent was obtained from all participants for whom identifying information is included in this article.

The study was approved (Pr. Nr: 012/2019) by the Regional Ethical Committee of Liguria.

### 2.2 Procedure

We used participant observation, as conceptualized in research in Infant Observation,^12^, ^13^ supported by video recordings.

A trained psychologist was assigned to each individual. Every month, they conducted two 90‐minute observational sessions over a defined period of 18 months.

### 2.3 Data collection

The different types of harm, developed from previous publication,^14^ involving other patients, were grouped into four categories:

1. *Self‐inflicted harm*: an action causing physical harm to the individual's body (eg, biting, etc.).
2. *Harm/damage to other people/objects*: an action causing physical harm to other people or damaging objects inside the peri‐personal space (eg, biting or hitting people, throwing objects).
3. *Harm to communication in progress*: an act of communication causing confusion, misunderstanding, or effects contrary to those desired (eg, not responding, giving the wrong answer, screaming, insulting).
4. *Harm to an activity in progress*: an action that harms the caregiver's activity with the child, preventing correct motor synergies to achieve the goal (eg, feeding or getting dressed), or harming the child's own performance (eg, cancelling/deleting the work just done on the PC).

Each recorded LNB was analyzed according to the following descriptive criteria:

1. *Environment*: in which context the LNB occurred.
2. *Interruption*: LNB was stopped in time, avoiding negative consequences.
3. *Repetition*: LNB was repeated after a short time (30 seconds).
4. *Antecedents*
  - Emotional state of the child: calm or agitated.
  - Situational qualitative categories, not mutually exclusive, describing the environmental conditions before each LNB:
    - *Behavior elicitation*: an unwanted or harmful behavior prompted, named, or implied.
    - *Change of position*: the individual is moved from one spatial position to another.
    - *Opportunity for harm*: a stimulus representing a risk in the space within the child's reach *Pressure to perform*: an environmental expectation with respect to performing an action.
    - *Urgency/confusion*: the individual is challenged by multiple stimuli in an urgent and unclear way.
    - *Emptiness/boredom*: the individual is not engaged in any activity or stimulated by the environment.
    - *Other*: none of the above categories are satisfied.
5. *Consequences* for the child
  - Psychophysical consequences:
    - Presence of pain according to physiological behavioral scales used in developmental age^15^, ^16^
    - Increase in anxiety according to physiological behavioral scales used in developmental age^17^
  - Emotional reactions following LNB, nonmutually exclusive: suffering, shock, sorrow, anger, and satisfaction.
    By the term “satisfaction” we mean the feeling of pleasure gained from self‐harm. This presupposes pain misperception. Finding such reaction in patients would support the still widespread opinion that painful stimuli for the general population may be pleasant for patients, or at least that the secondary manipulative advantages (such as focusing general attention) may be preferred over physical pain, given the relative or absolute insensitivity to pain.
6. *Management* by the caregiver
  - Physical or psychoeducational and relational strategies grouped into nonmutually exclusive categories.
    - Physical restraint: contact, restriction, restraint aids (eg, sleeves, dental guards, bands, gloves, etc.).
    - Moving person/object: removing the individual from the source of potential harm.
    - Asking for/receiving help from other people.
    - Verbal/nonverbal reassurance: eg, smiling and caressing.
    - Distraction.
    - Irony: joking in a mild and good‐natured way.
    - Scolding: verbal admonishment, sarcasm, or ignoring impassively.
    - Recognizing the LNB without making too much of it and giving the child time in a caring way
  - Emotional reaction of the caregiver at the time of onset of LNB: calm or agitated.

### 2.4 Statistical Analysis

We used Microsoft Office Excel software to collect data and calculate percentages.

In Table 4A, the correlation between the “Management” and the “stop the crisis” categories are reported. Values are included between −1 and 1, and were calculated with the Seaborn Python library and the heat‐map function. Positive values represent efficacious management. Values close to 0 indicate lack of effectiveness, and negative values represent counterproductive effect of the management.

## 3 RESULTS

We considered the operational concept of “Lesch‐Nyhan Behavior” (LNB), that is, any compulsive, ego‐dystonic action, with an unusual pattern of movement in terms of strength, speed, and precision, causing harm to the individual. Every LNB was extrapolated from the observational material collected in written reports and discussed in‐group.^12^

A series of 292 LNBs was gathered from the reports of 60 sessions for over 90 observation hours.

Table 1 shows the frequency of each kind of harm and their repetition rate. Only 27% of all LNBs are repeated, and most of them are *harm to an activity in progress*.

**Table 1:** Frequency and repetition rate of each type of harm/damage

	Self‐inflicted harm	Harm/damage to other people/objects	Harm to communication taking place	Harm to an activity taking place
Nonrepeated LNBs	84%	94%	88%	60%
Repeated LNBs	16%	6%	12%	40%

*Note*: The type of harm at highest risk of reiteration is that to an activity in progress. Percentages are calculated on the total number of observations for each harm/damage category.

### 3.1 Mood and Situational antecedents

Despite affected children are known to display poor mood control, no additional drugs helping this aspect are in their daily schedule. To be secured by constraints, which they firmly require, is the best help for mood control. However, as shown in Table 2A, the child's mood before an LNB event does not seem to be significant in self‐inflicted harm and in harm to an activity in progress, while it is often calm before harm to communication and in harm/damage to other people/objects.

**Table 2:** Antecedents

A. Antecedent patient mood in the different types of harm/damage				
Previous mood^a^	Self‐inflicted harm	Harm/damage to other people/objects	Harm to communication taking place	Harm to an activity taking place
Calm	49%	84%	76%	52%
Agitated	51%	16%	24%	48%

B. Situational antecedents in the different types of harm/damage				
Antecedents^b^	Self‐inflicted harm	Harm/damage to other people/objects	Harm to communication taking place	Harm to an activity taking place
Behavioral evocation	29%	9%	20%	30%
Change of position	7%	0%	2%	19%
Pressure to perform	29%	13%	85%	61%
Opportunity	56%	97%	0%	11%
Emptiness/boredom	7%	3%	2%	4%
Urgency/confusion	24%	3%	22%	11%
Other	17%	0%	7%	11%

*Note*: Percentages are calculated on the observations' total number for each harm/damage category.

a Despite LND children are considered to display poor mood control, the antecedent mood before an LNB is more frequently calm than agitated.

b The simple opportunity is the most frequent situational antecedent in case of self‐injurious behavior and harm to other/object. The pressure to perform is always badly tolerated and frequently predisposing to harm to communication and to an activity in progress.

The most common situational antecedent (Table 2B) in the case of *self‐inflicted harm* was the mere opportunity to perform it.

Eliciting a possible way of getting hurt can also trigger self‐injury.

Pressure to perform, for example, explicit request not to bite himself could rather be a trigger.

In *harm/damage to other people/objects* in the peri‐personal space, the environmental opportunity is overwhelming. Occasionally, pressure to perform is also recorded as recalling dangerous behaviors.

In *harm to communication in progress*, the most frequent antecedent is pressure to perform, followed by urgency and confusion and behavioral elicitation, for example, presenting dichotomous questions also verbalizing the unwanted alternative.

In *harm to an activity in progress*, the most frequent antecedent is pressure to perform, for example, requesting cooperation during handling manoeuvres. Behavioral elicitation and position change follow.

### 3.2 Pain, anxiety, and emotional consequences

Pain (Table 3A) is a normal reaction to self‐inflicted harm, explaining why increased anxiety is the typical psychophysical reaction after any LNB (Table 3B).

**Table 3:** Reactions

A. Psychophysical reactions of pain^a^					
Pain	Self‐inflicted harm	Harm/damage to other people/objects	Harm to communication taking place	Harm to an activity taking place	
No	2%	95%	100%	99%	
Yes	98%	5%	0%	1%	

B. Psychophysical reactions of anxiety^b^					
Anxiety increase	Self‐inflicted harm	Harm/damage to other people/objects	Harm to communication taking place	Harm to an activity taking place	
No	3%	25%	15%	10%	
Yes	97%	75%	85%	90%	

C. Emotional reactions^c^					
Emotional reactions	Self‐inflicted harm	Harm/damage to other people/objects	Harm to communication taking place	Harm to an activity taking place	
Anguish‐terror	49%	38%	7%	53%	
Shock‐disconcert	37%	84%	56%	48%	
Regret	29%	78%	68%	78%	
Anger	11%	6%	10%	32%	
Satisfaction	0%	0%	0%	0%	

D. Emotional reactions in repeated LNBs^d^					
Emotional reactions	Self‐inflicted harm	Harm/damage to other people/objects	Harm to communication taking place	Harm to an activity taking place	Total of LNBs
Anguish‐terror	82%	50%	0%	73%	69%
Shock‐disconcert	27%	50%	40%	50%	46%
Regret	27%	50%	40%	85%	73%
Anger	18%	50%	0%	50%	42%
Satisfaction	0%	0%	0%	0%	0%

*Note*: Percentages are calculated on the observations' total number for each harm/damage category.

a Reaction of pain is observed only in case of self‐inflicted harm.

b Increase in anxiety is the common reaction to every LNB.

c Emotional reactions associated with different types of harm/damage can widely vary, but satisfaction has never been observed.

d The most frequently observed emotional reaction to self‐inflicted harm is terror.

The most frequent emotional reaction (Table 3C) is regret, which is prevalent in harm/damage to other people/objects and harm to an activity in progress.

Shock is reported in half of the total sample, prevalent in harm/damage to other people/objects, frequent in harm to communication and to an activity in progress, and also in self‐inflicted harm.

Anguish is frequent in the case of harm to an activity in progress, self‐inflicted harm, and harm/damage to other people/objects.

Anger is mainly recorded in harm to an activity in progress and is rare in the other categories.

No satisfaction was ever recorded after any of the 292 LNBs.

In repeated LNB (Table 3D), anger, anguish, and regret increase.

### 3.3 Caregiver management and her/his mood

The most effective strategies to stop (Table 4A) self‐inflicted harm are moving object/individual, physical restraint, and distraction.

**Table 4:** Management

A. Correlation between management actions and their effectiveness^a^				
Management	Self‐inflicted harm^b^	Harm/damage to other people/objects	Harm to communication taking place	Harm to an activity taking place
Moving object/patient	0.45	0.37		0.08
Distraction	0.26		0.20	0.28
Irony	0.05	−0.06	0.43	0.17
Physical containment	0.32	0.03		0.16
Verbal reassurance	−0.17	0.09	0.09	0.13
Asking/receiving help				−0.10
Ignoring LNB	−0.46	−0.39	0.14	−0.30
Scolding	−0.24	−0.54	−0.38	−0.16

B. Caregiver's mood during management^c^				
Caregiver's mood	Interrupts	Does not interrupt	Total of LNBs	
Upset	29%^d^	71%	27%	
Calm	52%	48%	73%	

a Management effectiveness in interrupting or preventing the LNB repetition. For each harm category positive values represent efficacious management; values close to 0 indicate lack of effectiveness, and negative values represent counterproductive effect of the management.

b Removal of the object from patient's reach is the most efficacious strategy in case of Self‐Inflicted harm and harm to other/objects, while distraction and irony are the most efficacious ones in case of harm to communication and activity in progress. Ignoring the LNB and scolding, instead, almost always seriously worsen the self‐harming behavior.

c Calmness is preferable, but not sufficient to prevent the LNB.

d The percentages are calculated on the total number of LNBs observed.

The only strategy in the case of harm/damage to other people/objects seems to be moving object/individual.

The most useful strategies in both harm to communication in progress and harm to an activity in progress are irony and distraction, and physical containment for the latter.

The coefficient of scolding is negative in all types of harm, especially in harm to others/objects.

Regardless of the strategy used, the management approach of an agitated or upset caregiver seems unlikely to be effective; on the other hand, calmness alone does not necessarily stop the LNB underway (Table 4B).

## 4 DISCUSSION

The large amount of data collected through the participant observation method allowed extending the range of expressions of self‐harm in LND. Every LNB entails negative consequences for the individual directly (harm to his own body) or indirectly (harm/damage to other people/objects, to communication, and to activity in progress). The low recurrence frequency recorded in our sample (27%) and the low frequency of self‐inflicted harm (24%) is probably due to the physical restraint system and to the constant presence of an attentive caregiver.

Unlike other neurodevelopmental or psychiatric disorders, self‐injury in LND does not occur in response to agitation or boredom, but stress may be an aggravating factor.

The inconsistency of the hypothesis of pain insensitivity was confirmed, as previously reported.^18^, ^19^, ^20^ None of the 292 reported LNB made the children self‐satisfied and the most frequent emotional reactions are not only regret and anguish, but also shock, confirming the absence of intention^19^ and indicating how LNB can be unexpected and intrusive for the individual. Patients often request help for their own or others safety, confirming that it is not possible to knowingly stop LNB^20^ and that physical restraints are experienced as reassuring protection.

In our sample, the most frequent and recurrent (40%) LNB is *harm to an activity in progress*. This type of harm comprises opposite movements, probably the “oppositional behavior” described in literature as typical of LND is possibly due to the difficult management of complex cares (removal of restraints, change of position, bathing, etc.).^11^, ^21^, ^22^

The most dangerous and feared LNB, *self‐inflicted harm* is second in frequency. If not interrupted in time, it inevitably produces a psychophysical pain reaction. The mere presence of the opportunity for harm in the environment often triggers self‐harm in a number of ways and types. Only constantly monitored and adjusted restraints may have a chance of reducing danger and distress, though unpredictable LNBs may occur anyway. Self‐mutilation, often described as a typical symptom of LND, appears as the extreme consequence of the failure of managing and preventing reoccurrence. The prevalent emotional reactions are anguish/terror and also shock, further underscoring the specificity of self‐harm in LND.

The modest frequency of *harm to communication in progress*, mainly described as difficulties to start speaking, or as giving a wrong answer, is partly due to the young age of two of the three subjects included in the study. The unwanted answer is often described as loud and sudden, given with minimal latency, and appears similar to an unwanted action. Regret and shock are the most frequent emotional reactions, suggesting that also the swear words and insults ascribed to “verbal aggression” in literature^5^, ^22^ are unintentional.

*Harm/damage to other people/objects* is the least represented type of harm (11%), and features three highly significant parameters: the antecedent calm mood (84%); the consequent shocked reaction (84%); and an opportunity for harm/damage in the peri‐personal space (97%).

The univocal trend of these data and the minimal latency of the gesture emission from the opportunity to reach the person/object suggest a lack of intention excluding a cognitive evaluation. Therefore, the term *aggression* 21, 22 appears inappropriate for this type of LNB, regardless of the severity of consequences.

The LNBs managing strategies used by the caregivers were difficult to study and submit to a quantitative analysis. Caregivers often used more than one strategy toward a single LNB. Nevertheless, a set of strategies was defined and preliminary data about their effectiveness were collected. In cases of self‐inflicted harm and harm/damage to other people/objects, the first strategy for safety is moving away the individual from the source of danger. Indeed, it was often difficult to decide whether to record certain LNB as self‐harm or harm to others, because one single action was able to provoke both. “Letting it go compassionately,” probably decreasing the pressure to perform, only seems to work in the case of harm to communication. Likewise, irony, with its downplaying effect, seems to help. Distraction presents a positive correlation coefficient, especially in the case of harm to an activity in progress, but also in self‐harm. “Scolding or impassively ignoring” an LNB is never effective and can even become very dangerous in the case of self‐harm.

Caregivers describe “crises” during which the individual repeats a specific LNB many times and cannot stop till someone helps him. New LNBs can enter the individual's personal repertoire, sometimes occasionally, while well‐managed ones decrease. Further studies, based on larger numbers of patients, are required to feature the best array of LNBs' management strategies.

## 5 CONCLUSION

This paper suggests a broader view of the neurobehavioral disorder in LND and proposes a new description of LNB, wider than the classical SIB,

The breakdown of harm into four different types described in this paper is useful for analyzing the environmental risk factor, and provides pragmatic suggestions for the prevention of LNBs and the management of their recurrences. Caregivers and operators should be aware of and trained in recognizing the different types of LNB in order to find the better way to manage the behavior and communicate with patients.

## CONFLICT OF INTEREST

Anna Bozano, Alessandra Spessa, and Francesca Valeriani received a research grant from the “LND Famiglie Italiane” NPO. The same NPO paid travel expenses for research meetings to all authors.

## AUTHOR CONTRIBUTIONS

B.A. followed one patient and gave the main contribution in analyzing the data and writing the article. Schiaffino A. gave a great contribution in analyzing the data and writing the article. Spessa A. followed one patient, wrote the reports, and discussed results. V.F. followed one patient, wrote the reports, and discussed results. M.R. provided the statistical support. M. V. greatly helped in discussing results and writing the article. D.D. organized the project, wrote the article, and is the corresponding author of the work.

## ETHICAL COMMITTEE APPROVAL

The study was approved (Pr. Nr: 012/2019) by the Liguria Region Ethical Committee. (Ospedale Policlinico San Martino, Largo R. Benzi 10‐16 132 Genova).

## PATIENT CONSENT STATEMENT

After being carefully informed about the project, parents signed a written consent, available upon request. The study was approved (Pr. Nr: 012/2019) by the Regional Ethical Committee of Liguria. All procedures followed were in accordance with the ethical standards of the responsible committee on human experimentation (institutional and national) and with the Helsinki Declaration of 1975, as revised in 2000.
